# Advanced Stage Hepatic Fibrosis With Normal Liver Chemistries: A Case Report

**DOI:** 10.7759/cureus.40732

**Published:** 2023-06-21

**Authors:** Haley Gallaher, Sumyra Mehkri

**Affiliations:** 1 Medicine, Saint James School of Medicine, The Quarter, AIA; 2 Family Medicine, ASAS Health Valley Regional Diagnostics, Edinburg, USA

**Keywords:** alanine aminotransferase (alt), aspartate aminotransferase (ast), gamma-glutamyl transpeptidase (ggt)-to-platelet ratio, alkaline phosphatase (alp), liver function tests (lfts)

## Abstract

This is a case report on a patient with biopsy-proven stage 2 hepatic fibrosis who routinely has had normal liver chemistries. This is important because liver chemistry tests are the standard physicians use to assess general liver health.

There is not an abundance of research on patients with advanced hepatic fibrosis who have normal liver chemistries, but the research that is available all agree that liver biopsy is the better way to get a clear picture of the disease state of the liver. Biopsies of the liver are not as easily accessible or as cost-effective as simple blood work though. Patients are also less likely to agree to undergo a procedure than to get blood drawn. These are all obstacles in regard to changing the standard in determining liver health from laboratory work to liver biopsies, in addition to liver chemistries.

This case report aids in the research already available on how normal liver chemistries should not automatically rule out hepatic fibrosis. This patient proves that fibrosis can be happening, and the blood tests are not reflecting that change. Due to this, further research and investigation on liver fibrosis and normal liver chemistries should be done.

## Introduction

This is a case report on a patient with biopsy-proven stage 2 hepatic fibrosis who had routine liver chemical tests that were consistently within normal limits. Because chronic fibrosis can lead to cirrhosis, which puts patients at a higher risk for hepatocellular carcinoma, portal hypertension, hepatic encephalopathy, and a host of other ailments, it is important for clinicians to be able to identify liver injury and damage [1]. As of June 2022, liver chemical tests are the standard that physicians use to regularly assess liver health. Liver chemistries can be normal over time if there is enough healthy liver tissue. Therefore, it is easy for hepatic fibrosis to go undetected with routine laboratory work if the fibrosis is not extensive [2]. It is also important to note that liver chemistries can be abnormal in patients with healthy livers as well.

The liver is composed of hepatocytes and non-parenchymal cells, such as hepatic stellate cells. Once the liver is damaged, the hepatic stellate cells, located in the space of Disse, are activated to produce an extracellular matrix (ECM), promoting angiogenesis via the stimulation of vascular endothelial growth factor (VEGF). The overextension of the extracellular matrix and vascular endothelial growth factor causes a pro-fibrotic state in the liver. This excess extracellular matrix leads to basement membrane replacement by thick scar tissue called fibrosis [3]. This imbalance between synthesis and degradation of ECM results in hepatic fibrogenesis. Chronic progressive fibrogenesis leads to stage 4 fibrosis, also called cirrhosis [1].

Liver chemistries are blood tests that help determine the health of the liver. They measure the levels of different proteins and enzymes that are released when there is an injury to the liver. These include alanine transaminase (ALT), aspartate aminotransferase (AST), alkaline phosphatase (ALP), and gamma-glutamyl transpeptidase (GGT). ALT is an enzyme only found in the liver, so it is specific to liver injury. AST is an enzyme that is found not only in the liver but also in skeletal muscle, cardiac muscle, and renal and brain tissues. ALP is found throughout the body but is primarily found in the hepatobiliary tract and bone. GGT is found in the pancreas, renal tissues, biliary tract, and liver. Albumin and prothrombin time are markers used to assess hepatocellular function. Albumin is a plasma protein, and its synthesis happens exclusively in the liver [4]. Prothrombin time measures the clotting factors 1, 2, 5, 7, 9, and 10, which are synthesized in the liver. Significant hepatocellular injury can cause prolonged prothrombin time [5].

Hepatic fibrosis that is not due to any offending agent is harder to identify due to the lack of underlying disease or risk factor that would provoke suspicion of liver damage. Because of this, it can take a longer time to reach a diagnosis of hepatic fibrosis. Eventually, hepatic fibrosis can progress to cirrhosis, which can be irreversible once identified due to the chronicity of the damage. Due to the higher potential of damage permanence in hepatic cirrhosis, it is of great importance to be able to identify liver injury and damage so function can be maintained [6].

This case proves that liver chemistries solely are not a complete picture of liver health. This patient, with no known offending agent or genetic hepatic conditions, went in for cholecystectomy and had a wedge biopsy done of her liver simultaneously, which is how she was diagnosed with stage 2 liver fibrosis.

## Case presentation

The patient is a 66-year-old G3P3 Hispanic female with a body mass index (BMI) of 31.4 kg/m^2^. She had been going to her family physician since 2016 for epigastric and right upper quadrant pain. Since 2016, her liver chemistries have been within normal limits, and all imaging was only suggestive of cholelithiasis without cholecystitis. During this time, the patient declined a surgical consult, stating that she would not have surgery even if it was recommended and that her symptoms were not severe enough for surgery.

The patient has an extensive past medical history of human papillomavirus infection, cystocele, rectocele, lumbar disc herniation (2015), hyperlipidemia, osteoporosis, uterine leiomyoma, vitamin D deficiency, mild tricuspid regurgitation (2016), decompensated congestive heart failure New York Heart Association stage 2-3, urge and stress incontinence (2018), uterine leiomyoma, ovarian cyst (2019), secondary hypothyroidism (2020), and osteoarthritis of the carpometacarpal joint of the right thumb (2022).

The patient’s past surgical history includes tubal ligation in 1987, breast lumpectomy in 2014, dilation and curettage, ultrasound-guided needle biopsy of the right axillary lymph node indicating follicular lymphoma low-grade diffuse pattern, and cholecystectomy in 2022.

Her current medications are diclofenac potassium 50 mg a day for low back pain, Ditropan XL 5 mg extended-release daily for mixed incontinence, Flonase allergy relief 50 mcg/actuation nasal suspension, loratadine 10 mg a day for allergies, megestrol acetate 40 mg twice a day for postmenopausal hormone replacement, and Voltaren 1% external gel every 12 hours as needed for pain. The patient has no history of alcohol, tobacco, or drug use. Family history includes diabetes mellitus in the mother.

When the patient came to the clinic in February 2022 complaining again of abdominal pain, a right upper quadrant sonogram was performed. The sonogram showed gallstones without bile duct dilation and a normal liver appearance. Laboratory results were also done at this time and showed normal liver chemistries, the same as they have been throughout the years. She states this time that the right upper quadrant pain just comes and goes and is present when she lies down on the right side. She denied any nausea or vomiting, diarrhea, or constipation at that time. She was advised to refrain from fatty and fried foods, follow a strict bland diet, and see a surgeon as soon as possible.

That next week, the patient was seen by the surgeon who ordered a computed tomography (CT) scan of the abdomen due to atypical presentation of no pain alterations with eating and no nausea, vomiting, or bowel changes. The CT results aligned with the previous sonogram results suggestive of cholelithiasis. The patient agreed to surgery for the removal of her gallbladder at that time. The cholecystectomy was scheduled for the following month, and the patient was instructed to follow a strict bland diet avoidant of fried, fatty, greasy, and spicy food until then.

**Figure 1:**
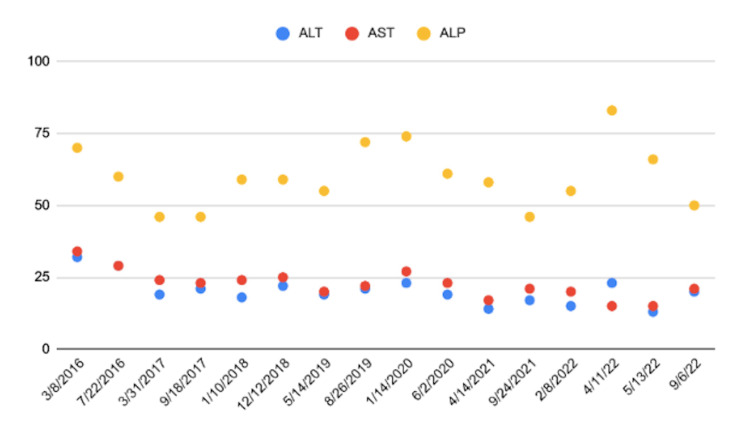
Patient’s Liver Function Test Results From 2016 to 2022 The X-axis depicts the dates of laboratory results of the liver chemistries from the patient’s initial visit for abdominal pain to her most recent laboratory work. The Y-axis depicts the liver function test results. The blue dots depict ALT, the red dots depict AST, and the yellow dots depict ALP. The values that are considered normal for adult females are 19-25 units/L for ALT, 9-32 units/L for AST, and 30-100 units/L for ALP. ALT: alanine transaminase, AST: aspartate aminotransferase, ALP: alkaline phosphatase

The operative report showed that a 2-centimeter (cm) wedge biopsy of the liver and a hysterectomy with dilation and curettage were done simultaneously with the cholecystectomy. The findings were consistent with a chronically inflamed gallbladder with “a lot of stones and sludge.” The liver biopsy showed possible cirrhotic liver. The surgeon referred the patient to a gastroenterologist for possible cirrhotic liver findings on wedge biopsy. During this time, the patient’s liver chemistries were continuing to be grossly within normal limits (Figure 1).

The patient was seen by the gastroenterologist in July 2022 for the liver wedge biopsy results suggestive of a possible cirrhotic liver. The final pathology report for the liver biopsy was dictated as “fatty liver disease, nonalcoholic fatty liver disease (NAFLD) activity score (NAS): 5 points (steatosis: approximately 30% (grade 1)), inflammation: lobular inflammation: <2 foci/20 × field (grade 1), balloon degeneration: 0 (none), stage 2-3 (perivenular, portal fibrosis and rare bridging fibrosis).” A nonalcoholic steatohepatitis (NASH) FibroSURE serum laboratory draw was done at the time (Table 1). NASH FibroSURE is an assessment of severity in patients with nonalcoholic fatty liver disease. It is not FDA-approved for diagnosis, but it is used to help determine the status of fatty liver disease in a noninvasive way. The gastroenterologist also did laboratory tests for mitochondrial antibody screen and titer as well as alpha-1-antitrypsin testing to look for primary biliary cholangitis and alpha-1-antitrypsin deficiency, respectively, but they came back negative.

**Table 1:** NASH FibroSURE Results The quantitative results of 10 biochemicals in combination with age, gender, height, and weight are analyzed using a computational algorithm to provide a quantitative surrogate marker (0.0-1.0) of liver fibrosis (Metavir F0-F4), hepatic steatosis (0.0-1.0, S0-S3), and NASH (0.0-0.075, N0-N2). The absence of steatosis (S<0.38) precludes the diagnosis of NASH. NASH: nonalcoholic steatohepatitis, GGT: gamma-glutamyl transpeptidase, ALT: alanine aminotransferase, SGPT: serum glutamic pyruvic transaminase, PSP: polysaccharide peptide, AST: aspartate aminotransferase, SGOT: serum glutamic-oxaloacetic transaminase

Test	Current Result and Flag	Units	Reference Interval
Fibrosis Score	0.49 (High)		0.00-0.21
Fibrosis Stage	F2 (Bridging Fibrosis With Few Septa)		
Steatosis Score	0.43 (High)		0.00-0.30
Steatosis Grade	S1 (Mild Steatosis)		
NASH Score	0.50 (High)		0.25
NASH Grade	N1 (Borderline or Probable NASH)		
Alpha 1-Macroglobulins, Qn	291 (High)	mg/dL	110-276
Haptoglobin	111	mg/dL	37-355
Apolipoprotein A-1	126	mg/dL	116-209
Bilirubin, Total	0.6	mg/dL	0.0-1.2
GGT	21	IU/L	0-60
ALT (SGPT) PSP	21	IU/L	0-40
AST (SGOT) PSP	25	IU/L	0-40
Cholesterol, Total	134	mg/dL	100-199
Glucose, Serum	95	mg/dL	65-99
Triglycerides	75	mg/dL	0-149

Viral hepatitis A, B, and C antigens were also tested, and the hepatitis A antibody total came back as reactive, indicating a prior hepatitis A infection. Some complications of hepatitis A can include cholestatic hepatitis, relapsing hepatitis, and autoimmune hepatitis [7,8]. The smooth muscle antibody screen and titer also came back positive, but a weak positive. A weak positive is considered 1:20-1:80, and this patient’s value was 1:20. With a weak positive, repeat testing was recommended to be done in 2-4 weeks. A positive smooth muscle titer indicates that the patient likely has autoimmune hepatitis. Autoimmune hepatitis refers to an autoantibody attacking smooth muscle cells in the liver [7]. This weak positive result is peculiar, as ANA are the most common circulating antibodies in autoimmune hepatitis. It is important to note that smooth muscle antibodies are considered more specific than ANA for autoimmune hepatitis, although they are less prevalent. The patient will get repeated smooth muscle antibody titers, but at this point, the patient does not meet the diagnostic criteria for autoimmune hepatitis. The criteria needed to make this diagnosis is a minimum of one elevated serum aminotransferase at least two times the upper limit of the reference range, a minimum of one positive laboratory test (increase total IgG or gamma globulin levels), and/or serologic markers (ANA and ASMA at a titer of at least 1:40), and exclusion of other diseases (viral hepatitis, drug-induced liver injury, and alcoholic liver disease). A liver biopsy can make a confirmatory diagnosis if histology shows interface hepatitis and/or predominantly lymphoplasmacytic infiltrates [4,2].

## Discussion

As of January 2023, the Centers for Disease Control and Prevention states that approximately 4.5 million adults in the United States of America are diagnosed with liver disease [9]. This information illustrates the importance of identifying liver disease early in the disease course as early detection allows for earlier intervention. Early treatment will reduce the severity of the imbalance between synthesis and degradation of the extracellular matrix leading to chronic fibrosis [2]. This case illuminates the importance of not relying solely on liver chemistries to give a complete picture of liver health. It is evident here that a patient can have consistently normal liver chemistries but have liver damage going undetected. Chronic liver disease is a common cause of morbidity and mortality in the United States. According to the Centers for Disease Control and Prevention, chronic liver disease and cirrhosis ranked number 9 as the overall cause of death [9]. This knowledge of chronic liver disease and cirrhosis being such a major cause of mortality further supports the idea of implementing liver biopsies in diagnostic plans for patients with questionable liver disease in the setting of normal liver chemistries. The literature shows that it is vital to be able to detect and treat liver damage as early as possible to prevent chronic fibrosis and eventually irreversible cirrhosis [5]. The severity of fibrosis determines the staging of actual liver disease; therefore, the longer the hepatocellular injury goes undetected, the more continuous damage there is to the liver, leading to cirrhosis [1]. As stated previously, it is more difficult to identify hepatic injury without any underlying disease or risk factors. Some differential diagnoses to rule out would be autoimmune hepatitis, viral hepatitis, hepatotoxin exposures such as medications and alcohol, metabolic and genetic disorders, and malignancy [10].

This case report is not without its limitations. The patient only speaks Spanish; therefore, it is not impossible that the language barrier could have impeded treatment, although an interpreter was used. Because of the language barrier and the use of an interpreter, there is a possibility of miscommunication, misinterpretation, and lack of understanding. Additionally, the patient was not willing to undergo additional testing, such as additional blood work for autoimmune titers, genetic workup, surgical consultation, or even additional imaging, at the start of her abdominal pain, potentially delaying the diagnosis of her stage 2 hepatic fibrosis.

## Conclusions

Like many things in medicine, screening and prevention are key, so it is important to further assess for liver damage, despite normal liver chemistries, in patients that give clinical suspicion for liver damage. This patient had liver chemistries checked routinely, with results always within reference ranges. The patient never reported any other signs or symptoms of liver disease, such as changes in her bowel habits or changes in appetite, other than ongoing abdominal pain. Clinically, there were never any signs to suggest liver disease, such as jaundice, anemia, palmar erythema, gynecomastia, caput medusae, edema, spider angiomas, or increased jugular venous pressure. In addition, she never experienced nausea, vomiting, or changes in appetite. Because of her atypical presentation and gallstones contributing to her abdominal pain, no further workup for liver health was deemed necessary. The liver wedge biopsy that was done at the time of her cholecystectomy showed stage 2-3 hepatic fibrosis. The patient will now be following up with a gastroenterologist, including getting repeat smooth muscle antibody laboratory tests. This case report shows that liver damage can occur in atypical presentations and normal laboratory results. Further case reports and research would be beneficial to uncover better ways of screening for liver health.
